# A G-protein-coupled receptor regulation pathway in cytochrome P450-mediated permethrin-resistance in mosquitoes, *Culex quinquefasciatus*

**DOI:** 10.1038/srep17772

**Published:** 2015-12-10

**Authors:** Ting Li, Chuanwang Cao, Ting Yang, Lee Zhang, Lin He, Zhiyong Xi, Guowu Bian, Nannan Liu

**Affiliations:** 1 Department of Entomology and Plant Pathology, Auburn University, Auburn AL 36849; 2 Genomics Laboratory, Auburn University, Auburn, AL 36849; 3 Department of Microbiology and Molecular Genetics, Michigan State University, East Lansing, MI 48824.

## Abstract

Rhodopsin-like G protein-coupled receptors (GPCRs) are known to be involved in the GPCR signal transduction system and regulate many essential physiological processes in organisms. This study, for the first time, revealed that knockdown of the rhodopsin-like GPCR gene in resistant mosquitoes resulted in a reduction of mosquitoes’ resistance to permethrin, simultaneously reducing the expression of two cAMP-dependent protein kinase A genes (PKAs) and four resistance related cytochrome P450 genes. The function of rhodopsin-like GPCR was further confirmed using transgenic lines of *Drosophila melanogaster*, in which the tolerance to permethrin and the expression of *Drosophila* resistance P450 genes were both increased. The roles of GPCR signaling pathway second messenger cyclic adenosine monophosphate (cAMP) and downstream effectors PKAs in resistance were investigated using cAMP production inhibitor Bupivacaine HCl and the RNAi technique. Inhibition of cAMP production led to significant decreases in both the expression of four resistance P450 genes and two PKA genes and mosquito resistance to permethrin. Knockdown of the PKA genes had shown the similar effects on permethrin resistance and P450 gene expression. Taken together, our studies revealed, for the first time, the role of the GPCR/cAMP/PKA-mediated regulatory pathway governing P450 gene expression and P450-mediated resistance in *Culex* mosquitoes.

Mosquitoes are known vectors of parasites and pathogens for a number of human and animal diseases. *Culex quinquefasciatus* is an important mosquito vector for transmission of pathogens that cause West Nile encephalitis, Saint Louis encephalitis, and lymphatic filariasis^1^. Mosquito vector control is an important component of the global strategy to control mosquito-associated diseases and insecticides are the most important weapon in the arsenal of those working to reduce vector-borne diseases and protect public health. Pyrethroids are currently the most widely used insecticides for the indoor control of mosquitoes worldwide and the only chemical recommended for the treatment of mosquito nets^2^, the main tool for preventing malaria in Africa. In the past, massive sprayings of insecticides greatly limited mosquito-borne diseases and even eradicated malaria in a few areas^3^. However, the development of resistance to insecticides, especially pyrethroids, in mosquito vectors has become a global problem and there has been a resurgence in mosquito-related diseases in many parts of the world^2^,^3^.

Among the mechanisms responsible for insecticide resistance, increasing the metabolism of insecticides into less harmful substances and facilitating insecticide excretion by cytochrome P450s are known to play critical roles in enabling insects to defend themselves against insecticides^4^,^5^. The up-regulation of P450 genes, resulting in increased levels of P450 proteins and P450 activities, has been firmly linked to the enhanced metabolic detoxification of insecticides and has been implicated in the development of insecticide resistance in a number of insects, including mosquitos^5^,^6^,^7^. Studies on P450 gene up-regulation or induction in response to xenobiotics have indicated that *cis-* or *trans*-acting regulators are involved in transcriptional regulation in insects^8^,^9^, suggesting that insecticide resistance is conferred via the interaction of regulatory factors/cis regulatory elements and resistance genes.

As part of effort to investigate the regulatory factors and pathways that are involved in insecticide resistance, the co-overexpression/interaction relationship of metabolic detoxification genes in *Cx. quinquefasciatus* has been investigated in our laboratory and revealed that multiple P450 gene interactions and their regulation play a key role in the development of insecticide resistance in mosquitoes^6^,^10^. Our previous studies^10^,^11^ have also identified a signaling transduction gene that encodes a G-protein coupled receptor (GPCR) and is co-up-regulated with P450 genes in resistant *Cx. quinquefasciatus*. GPCRs are known to be involved in recognizing extracellular messengers, transducer signaling to the cytosol, and mediating the cellular responses necessary for the normal physiology of organisms and have also been used as potential targets for therapeutic intervention in many human diseases^12^,^13^. Our further discovering of the function of GPCR genes in insecticide resistance revealed the involvement of GPCRs in the regulation of resistance P450 gene expression^14^. The current study extended our earlier efforts of understanding the function of the GPCR genes and their regulatory pathway(s) in insecticide resistance in *Cx. quinquefasciatus* mosquitoes. The effects of a rhodopsin-like GPCR and its downstream second messenger cAMP and effectors PKAs on insecticide resistance were tested. The GPCR/ cAMP/PKA signaling pathway in regulating resistant P450 gene overexpression was examined. The outcome of the study provides novel information concerning a GPCR/cAMP/PKA regulatory signaling pathway governing P450 gene expression and P450-mediated resistance in *Culex* mosquitoes.

## Results

### RNA interference (RNAi) functional study of up-regulated P450s in *Cx. quinquefasciatus*

**Figure 1:**
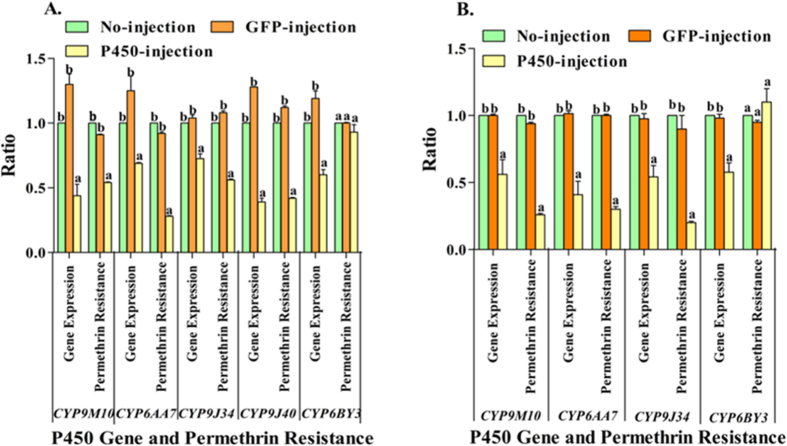
Cytochrome P450 genes in HAmCq^G8^ or MAmCq^G6^ associated with permethrin resistance. (**A**) mRNA levels of different P450 genes (up-regulated *CYP9M10, CYP6AA7, CYP9J34, CYP9J40*, or non-up-regulated *CYP6BY3* in resistant mosquitoes) were quantitatively tested by qRT-PCR at 3-days after dsRNA injection in HAmCq^G8^. Larva bioassay was conducted at 3-days after dsRNA of different P450 gene injection. LC_50_ was analyzed using standard probit analysis. The relative expressions of the P450 genes in P450-injected (yellow column) or GFP-injected (orange column) mosquitoes are shown relative to the expression in no-injected mosquitoes (green column). Permethrin resistance ratios were calculated by dividing the LC_50_ of the P450-injected or GFP-injection mosquitoes by the LC_50_ of the no-injected mosquitoes. (**B**) mRNA levels of 4 different P450 genes (*CYP9M10, CYP6AA7, CYP9J34*, or *CYP6BY3*) and permethrin resistance were conducted in MAmCq^G6^ following the same procedure as for dsRNA-injection in HAmCq^G8^. The results are shown as the mean ± S.E (n ≥ 3). Statistical significance is represented by P ≤ 0.05 in the level of the gene expression or resistance ratio among the P450-injected, GFP-injected and no-injected mosquitoes with different alphabet letters (a or b) using One-way ANOVA.

To functionally test the effect of P450 overexpression on permethrin resistance in *Cx. quinquefasciatus*, RNAi was used to knockdown four P450 genes known to be up-regulated in the resistant strains HAmCq^G8^ and MAmCq^G6^. Double-stranded RNA (dsRNA) generated from PCR amplicons of each P450 gene and a GFP control was micro-injected into ~1000 fresh grey embryos of each of the highly resistant HAmCq^G8^ and MAmCq^G6^ strains. Injected embryos were separated into two groups of ~360 each for RNA extraction and insecticide bioassay. RNAs were extracted and insecticide bioassays were conducted for each of the P450-injected, GFP-injected, no-injected and negative control (non-differentially expressed P450 gene, *CYP6BY3*) groups of second instar mosquitoes. The qRT-PCR results revealed a significant decrease of the P450 gene expression in all four of the up-regulated P450 dsRNA-injected mosquitoes compared with the control GFP-injected, no-injected (*P* values ranged from 0.019 to 0.001, Fig. 1) and negative controls, where P450 *CYP6BY3* was not up-regulated. The toxicity of permethrin to the mosquitoes injected with all four up-regulated P450 dsRNAs was significantly increased compared with the control GFP-injected and no-injected mosquitoes (*P* values ranged from 0.005 to 0.001, Fig. 1), but not in the negative control (injected with *CYP6BY3*) mosquitoes in both HAmCq^G8^ and MAmCq^G6^ (with *P* of 0.45and 0.379, respectively). The strong correlation between decreased P450 gene expression of all four genes and increased mortality levels to permethrin in P450 dsRNA-injected mosquitoes demonstrates the importance of these four P450 genes in insecticide resistance.

### GPCR gene expression in *Cx. quinquefasciatus*

**Figure 2:**
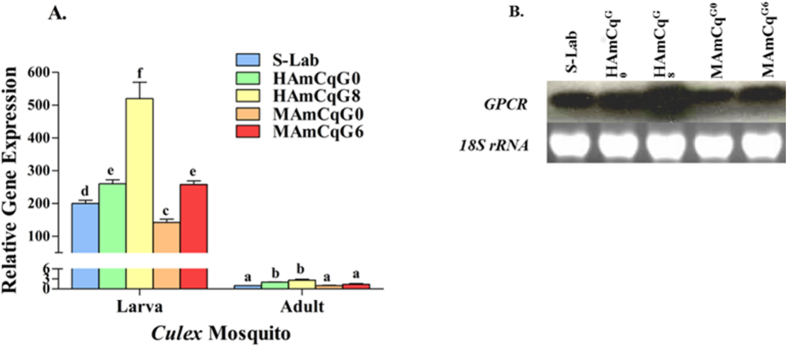
Expression pattern of the GPCR gene in the permethrin resistant and susceptible *Culex* mosquitoes. (**A**) mRNA was isolated from 4^th^ instar larvae and 3-day-old adults of different mosquito strains (S-Lab, HAmCq^G0^, HAmCq^G8^, MAmCq^G0^ and MAmCq^G6^). Relative expression of GPCR gene was tested using qRT-PCR. The relative level of gene expression shown along the Y axis is the ratio of the gene expression in each larva or adult of each strain compared with the gene expression in an S-Lab adult. The results are shown as the mean ± S.E (n ≥ 3). The significant difference is represented by P ≤ 0.05 in the level of GPCR gene expression among samples with different alphabet letters (a, b, c, d, or e) using One-way ANOVA data analysis. (**B**) Northern blot analysis shows the GPCR gene expression in the 4^th^ instar larva among different strains. The ethidium bromide stain of 18S ribosomal RNA in agarose gel is shown at the bottom. The gel has been run under the same experimental condition.

Full length rhodopsin-like GPCR cDNA was isolated from *Cx. quinquefasciatus* with 3′ and 5′ RACE using the specific primers designed for previous GPCR EST sequences^11^. The putative protein sequence of the rhodopsin-like GPCR (accession numbers: XP_001870286) deduced from the cDNA sequences shared 98% identity with *Cx. quinquefasciatus* GPCR GPROP13 (CPIJ020021). The expression pattern of the rhodopsin like GPCR gene was examined in 4^th^ instar larvae and 3 day old adults of the susceptible S-Lab strain, two field parental strains, HAmCq^G0^ and MAmCq^G0^, and two permethrin selected highly resistant strains, MAmCq^G6^ and HAmCq^G8^, using qRT-PCR. The results showed that the expression of rhodopsin-like GPCR gene was more than 200-fold higher in larvae than in adults (Fig. 2A), suggesting that the rhodopsin-like GPCR gene is developmentally regulated and more specifically expressed in larval mosquitoes. Comparisons of gene expression among larvae of five mosquito strains indicated that the rhodopsin-like GPCR was significantly overexpressed (2-fold, *P* < 0.001, or *P* = 0.004) in both HAmCq^G8^ and MAmCq^G6^, highly resistant selected lines, compared with their parental field strains, HAmCq^G0^ and HAmCq^G0^, respectively, suggesting that the level of rhodopsin-like GPCR expression increased following permethrin selection. The expression of rhodopsin-like GPCR in the field strain HAmCq^G0^ also showed significantly higher (1.4-fold, *P* = 0.031) expression compared with that in the laboratory susceptible strain, whereas, the field MAmCq^G0^ showed similar or lower expression compared to S-Lab (Fig. 2A). This suggests it may have been significantly down-regulated in MAmCq^G0^ compared with the other strains (*P* = 0.05) (Fig. 2A). To confirm these results, a northern blot analysis was performed and showed results that were consistent with those of the qRT-PCR study (Fig. 2B). Although the rhodopsin-like GPCR expression levels were considerably lower in adults compared with larvae, a significant increase in the level of expression was observed in both HAmCq^G8^ and HAmCq^G6^ compared with their parental field strains, HAmCq^G0^ and HAmCq^G0^, respectively, and S-lab.

### Functional study of the rhodopsin-like GPCR in *Cx. quinquefasciatus*

**Figure 3:**
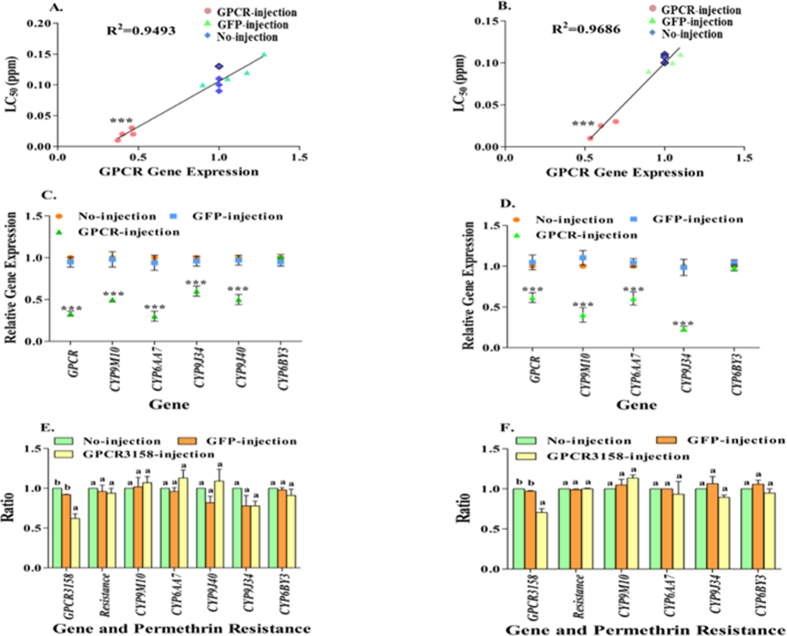
Relationship between GPCR gene, permethrin resistance and P450 gene expression. (**A**) The relative expression of GPCR gene presented along the X-axis was quantitatively tested at 3-days after GPCR-, GPF-, or No-injection for HAmCq^G8^ using qRT-PCR. The larva bioassay was performed at 3-days after dsRNA injection. The LC_50_ of GPCR-, GFP-, or No-injected mosquitoes shown along the Y-axis was calculated using probit analysis. The correlation between GPCR gene expression and permethrin resistance are shown as a logarithmic line and R^2^. (**B**) The correlation between GPCR gene expression and permethrin resistance with LC_50_ were performed in MAmCq^G6^ following the same procedure as for dsRNA-injection in HAmCq^G8^. (**C**) The different pattern expression of GPCR and 5 P450 genes (*CYP9M10, CYP6AA7, CYP9J34, CYP9J40*, or *CYP6BY3*) were tested at 3-days after dsRNA for GPCR-, GFP-, or No-injected HAmCq^G8^ mosquitoes using qRT-PCR. The relative gene expression ratio of the dsRNA-injected mosquitoes is shown relative to that in the no-injected mosquitoes. (**D**) The different patterns for the expression of GPCR and 4 P450 genes (*CYP9M10, CYP6AA7, CYP9J34*, or *CYP6BY3*) were tested in MAmCq^G6^ as for the dsRNA injected HAmCq^G8^. (**E**) A non-up-regulated GPCR gene, GPCR3158, served as the negative control. The different pattern expression of GPCR3158 and 5 P450 genes was quantitatively tested at 3-days after dsRNA of GPCR3158-, GFP-, and no-injected HAmCq^G8^ mosquitoes. A larva bioassay was conducted among the dsRNA-, and no-injected mosquitoes, and their resistance to permethrin expressed in terms of LC_50_. The ratio shown along the Y-axis is the ratio of gene expression or LC_50_ of dsRNA-injected to no-injected mosquitoes. (**F**) The relative expressions of GPCR3158, 4 P450 genes, or permethrin resistance ratio (LC_50_) were measured in MAmCq^G6^ as in HAmCq^G8^. The results are shown as the mean ± S.E (n ≥ 3). Statistical significance of the gene expression or resistance ratio among P450-, GFP- and no-injected mosquitoes was analyzed using One-way ANOVA. **P < 0.01, ***P < 0.001.

The rhodopsin-like GPCR dsRNA was injected into embryos of HAmCq^G8^ and MAmCq^G6^, followed by insecticide bioassay with larvae, to examine the levels of insecticide resistance to permethrin in the rhodopsin-like GPCR dsRNA-injected mosquitoes. Comparisons of relative gene expression and permethrin sensitivity between the rhodopsin-like GPCR dsRNA-injected HAmCq^G8^ and MAmCq^G6^ larvae and GFP-injected or no-injected controls revealed strong correlations (R^2^ = 0.9493, or R^2^ = 0.9686), with significant decreases (*P* < 0.001) in rhodopsin-like GPCR expression in both strains (Fig. 3A,B, X-axis) accompanied by increased toxicity to permethrin (*P* < 0.001, Fig. 3 Y-axis). To test our hypothesis further that rhodopsin-like GPCR signaling is involved in the up-regulation of P450 genes in mosquitoes, we tested changes in the expression of the four up-regulated P450 genes, *CYP9M10, CYP9J34, CYP9J40, CYP6AA7*, in the rhodopsin-like GPCR dsRNA-injected, GFP-injected, and no-injected mosquitoes. The results showed that suppression of the rhodopsin-like GPCR gene expression in the GPCR dsRNA-injected mosquitoes significantly decreased the expression of all four P450 genes (Fig. 3C,D, *P* < 0.001), while no significant changes in the P450 gene of *CYP6BY3* expression were detected in the rhodopsin-like GPCR-, GFP- and no-injected mosquitoes (Fig. 3C,D).

A parallel RNAi study was conducted on CPIJ003158 (accession: XM_001844694), a GPCR gene that showed no differential expression between resistant and susceptible mosquitoes. The expression of CPIJ003158 was significant decreased in CPIJ003158 dsRNA microinjected mosquitoes of both HAmCq^G8^ and MAmCq^G6^ strains, for both GFP-injected and no-injected mosquitoes (*P* = 0.007and *P* = 0.002, respectively) (Fig. 3E,F). Nevertheless, this significant decrease in the level of expression of the CPIJ003158 gene resulted in no significant change in the level of permethrin resistance in the mosquitoes compared with the no-injection and GFP injection (Fig. 3E,F) mosquitoes. These results demonstrate the important role of the up-regulated GPCR gene in permethrin resistance in *Cx. quinquefasciatus*, as suggested previously^11^. Moreover, the injection of the non-up-regulated GPCR gene (CPIJ003158) had no effect on either the P450 gene expression or the insecticide resistance.

### Function of the rhodopsin-like GPCR in transgenic *D. melanogaster*

**Figure 4:**
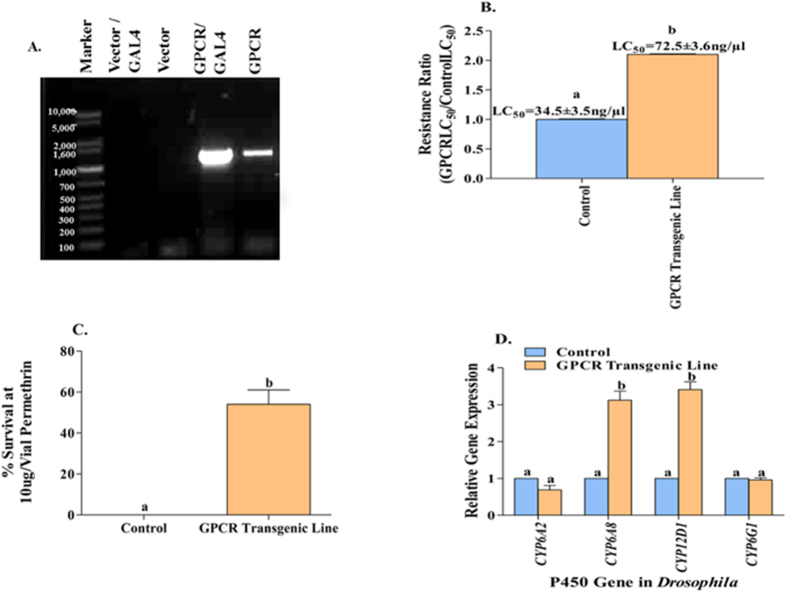
Effects of GPCR on permethrin resistance and P450 gene expression in *Drosophila melanogaster*. (**A**) The GPCR gene was amplified with primers *Drosophila* RhoF/ RhoR (1122 bp amplicon). Transcripts were proven for the GPCR gene in vector-transgenic lines (before or after cross with GAL4 line) and GPCR-transgenic lines (before or after cross with GAL4 line) using RT-PCR in gel electrophoresis, and the gel has been run under the same experimental condition (GPCR gene inserted in second autosome of *Dro. melanogaster*). (**B**) Control flies (vector/GAL4) and GPCR transgenic flies (GPCR/GAL4) were exposed to a series of doses (2, 4, 8, 10, 20 μg/vial), which caused mortality >0 and <100. LC_50_ was analyzed by probit analysis. The resistance ratio shown along the Y-axis was calculated by dividing the LC_50_ of the GPCR transgenic flies by the LC_50_ of the control. (**C**) Two-3 day-old control flies and GPCR transgenic flies were exposed to single doses of 10 μg permethrin/vial. The survival rate (%) was measured after 24 hrs exposed at 25 ± 2 °C. (**D**) The different patterns in the expression of 4 P450 genes (*CYP6A2, CYP6A8, CYP12D1, CYP6G1*) were performed in control and GPCR transgenic flies using qRT-PCR. The relative expression of the P450 genes in transgenic flies are shown relative to their expression in the control. The results are shown as the mean ± S.E (n ≥ 3). Statistical significance is represented by P ≤ 0.05 in the level of the gene expression or resistance ratio among the samples with different alphabet letters (a or b) using One-way ANOVA.

The rhodopsin-like GPCR gene was incorporated into the pUASTattB plasmid and transformed into the Drosophila 58A strain under control of the GAL4-UAS enhancer trap system (Rainbow Transgenic Flies, Inc., Camarillo, CA). *D. melanogaster* transformed with pUASTattB plasmid alone served as the control. RT-PCR amplification of the full length rhodopsin-like GPCR gene in both lines revealed that expression increased only in the GAL4-UAS line (Fig. 4A). The sensitivity of the *D. melanogaster* transgenic line to permethrin was characterized by comparing it to that of the control line. The transgenic line with the mosquito rhodopsin-like GPCR gene showed a 2-fold increase in permethrin tolerance compared to the control line in terms of the toxicity (LC_50_) to permethrin (Fig. 4B, *P* = 0.005). When adult female *D. melanogaster* were exposed to permethrin at a concentration of 10 μg/per vial *D. melanogaster* line with the rhodopsin-like GPCR gene had survival rates of 55% ± 7.8% compared 0% survival rates for the control *D. melanogaster* line after exposure to the same challenge (Fig. 4C). The capability of the mosquito rhodopsin-like GPCR gene to confer permethrin tolerance for *D. melanogaster* suggests that this up-regulated gene plays an important role in the resistance of mosquitoes to insecticides such as permethrin. We then characterized the expression of four *Drosophila* cytochrome P450 genes, *CYP6A2, CYP6A8, CYP12D1*, and *CYP6G1* (primers listed in Table S1), which had been reported to be involved in insecticide resistance in *Drosophila*^15^. Significant increases (*P* < 0.001) in the expression of CYP12d1 and CYP6a8 (~3.4-fold and ~3.1-fold, respectively) were found in the rhodopsin-like GPCR transgenic lines compared to the control lines (Fig. 4D).

### GPCR/ cAMP-dependent protein kinase A (PKA) pathway in regulation of P450 expression and function

**Figure 5:**
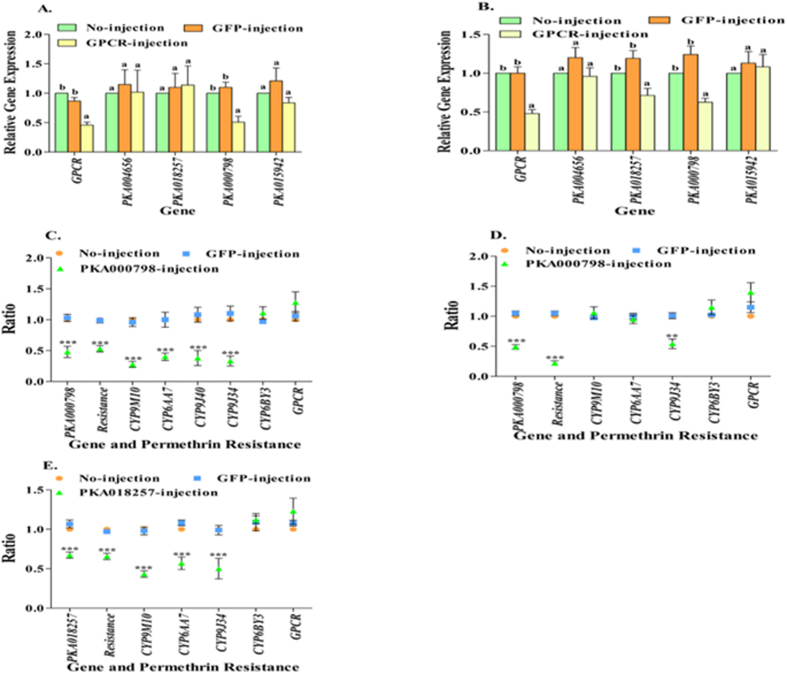
Association of GPCR/PKA pathway with permethrin resistance and P450 gene expression. (**A**) The different patterns in the expression of GPCR and 4 PKA genes (PKA004656, PKA018257, PKA000798, PKA015942) were performed at 3-days after dsRNA of GPCR-, GFP-, or no-injected HAmCq^G8^ mosquitoes using qRT-PCR. The relative expression ratios of genes in GPCR-, or GFP-injection mosquitoes are shown relative to those in no-injected mosquitoes. (**B**) The different patterns in the expression of GPCR and 4 PKA genes were tested in MAmCq^G6^ following the same procedure as for dsRNA-injection in HAmCq^G8^. (**C**) The relative expression of PKA000798, 5 P450 genes, or GPCR were performed at 3 days after dsRNA of PKA000798-, GFP-, or no-injection of HAmCq^G8^ mosquitoes using the qRT-PCR. The larva bioassay was conducted at 3-days after PKA-, GFP-, or no-injection. The ratio shown along the Y-axis is the ratio of the gene expression or LC_50_ of dsRNA-injection mosquitoes compared with no-injected mosquitoes. (**D**) The different patterns in the expression of PKA000798, 4 P450 genes, or GPCR, and permethrin resistance (LC_50_) were tested among dsRNA of PKA000798-, GFP-, and no-injected MAmCq^G6^ as for the HAmCq^G8^ mosquitoes. (**E**) The relative expression of PKA018257, 4 P450 genes, or GPCR, and permethrin resistance (LC_50_) were performed with dsRNA of PKA018257-, GFP-, and no-injected MAmCq^G6^ as for the PKA00798 knockdown in MAmCq^G6^ mosquitoes. The results are shown as the mean ± S.E (n ≥ 3). Statistical significance is represented by P ≤ 0.05 in the level of the gene expression among the samples with different alphabet letters (a or b) using One-way ANOVA. **indicates P < 0.01, ***P < 0.001.

We investigated the expression of a total of four PKAs (PKA000798, PKA018257, PKA004656 and PKA015942, with Accession numbers: XM_001842369.1, XM_001868347.1, and XM_001846139.1 and XM_001866281.1, respectively) that had been annotated in the genome of *Cx. quinquefasciatus* (VectorBase, Bioinformatics Resource for Invertebrate Vectors of Human Pathogens, https://www.vectorbase.org/search/site/camp%20protein%20kinase?species_category=%22Culex%20quinquefasciatus%22) in the rhodopsin-like GPCR dsRNA-injected HAmCq^G8^ and MAmCq^G6^ mosquitoes to identify key PKAs involved in the GPCR pathways known to be important for resistance. The results showed that one PKA gene, PKA000798, of the 4 tested exhibited significantly decreased expression levels in GPCR-knockdown HAmCq^G8^ mosquitoes (P < 0.001) compared with GFP-injected and no-injected mosquitoes (Fig. 5A). In the strain MAmCq^G6^, two PKA genes, PKA000798 and PKA018257, showed reduced levels of expression in GPCR knockdown mosquitoes (P < 0.001, P = 0.002, Fig. 5B), suggesting that PKAs are the downstream effectors of the GPCR/cAMP/ regulatory pathway. These results indicate that PKA(s) are indeed involved in the GPCR signaling pathways, but that different mosquito strains may use overlapping but strain-specific PKAs.

To confirm that PKAs act as GPCR effectors to regulate P450s, we next tested the involvement of PKA000798 and PKA018257 in permethrin resistance and P450 gene expression by injecting dsRNA of the PKA gene PKA000798 into embryos of both HAmCq^G8^ and MAmCq^G6^ mosquitoes and injecting dsRNA of PKA018257 into the MAmCq^G6^ embryos. The results showed significant correlations between decreases in the PKA000798 gene expression (~2.5-fold, P < 0.001 and ~2.0-fold, P < 0.001, respectively) and decreased levels of resistance in both strains (Fig. 5D) when comparing PKA-injected mosquitoes with GFP-injected and no-injected controls, functionally confirming the involvement of PKA000798 in permethrin resistance. Further examination of P450 and GPCR expression in the PKA-knockdown mosquitoes revealed a direct association between decreased levels of PKA000798 and reduced expression of all four P450 genes, *CYP9M10* (P < 0.001), *CYP6AA7* (P < 0.001), *CYP9J40* (P < 0.001), *CYP9J34* (P < 0.001), in HAmCq^G8^ compared with the GFP-injected and no-injected mosquitoes (Fig. 5C), while knockdown of the same PKA in MAmCq^G6^ impacted expression of only one P450 gene, *CYP9J34*, which decreased markedly (P = 0.01) (Fig. 5D). Knockdown of PKA018257 in MAmCq^G6^ also had an impact, decreasing *CYP9M10* (P < 0.001), *CYP6AA7* (P < 0.001), and *CYP9J34* (P < 0.001) (Fig. 5E). Nevertheless, knockdown of the PKAs in both strains had no effect on expression of the rhodopsin-like GPCR and *CYP6BY3* genes, suggesting that the four implicated P450 genes contribute to insecticide resistance through their overexpression, probably regulated by the GPCR and its downstream effector PKA signaling pathway.

**Figure 6:**
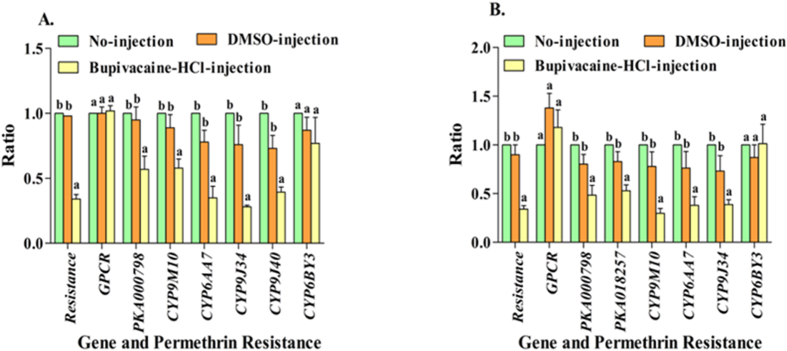
cAMP/PKA pathway associated with permethrin resistance and the expression of GPCR and P450 genes. (**A**) The larva bioassay was conducted at 3-days after cAMP-inhibitor (Bupivacaine-HCl, 40 μM)-, DMSO-, and no-injection in HAmCq^G8^ mosquitoes. The LC_50_ was analyzed by standard probit analysis, and the permethrin resistance ratio is the ratio of the LC_50_ of inhibitor-, or DMSO-injected mosquitoes expressed in relation to those for no-injected mosquitoes. The mRNA was isolated at 3-days after inhibitor-, DMSO-, and no-injected larva. The different pattern expressions of GPCR, PKA000798, and 5 P450 genes were tested in injected and no-injected mosquitoes using qRT-PCR. The ratio shown along the Y-axis is the ratio of the gene expression or LC_50_ of inhibitor-, or DMSO-injection mosquitoes compared with those in no-injected mosquitoes. (**B**) The permethrin resistance and relative gene expression patterns of GPCR, PKA000798, PKA018257, and 4 P450 genes were performed in MAmCq^G6^ as for the HAmCq^G8^ mosquitoes. The results are shown as the mean ± S.E (n ≥ 3). Statistical significance is represented by P ≤ 0.05 in the level of the gene expression or resistance ratio among the samples with different alphabet letters (a or b) using One-way ANOVA.

To investigate signaling between the rhodopsin-like GPCR and its PKA effectors, we examined the role of cAMP^16^, a messenger molecule commonly associated with GPCR pathways, in regulating P450 expression and influencing resistance in HAmCq^G8^ and MAmCq^G6^. We micro-injected 3^rd^ instar larvae of both HAmCq^G8^ and MAmCq^G6^ mosquitoes with DMSO-dissolved Bupivacaine HCl, the specific inhibitor of cAMP production, and DMSO alone and tested the permethrin toxicity and P450, PKA, and rhodopsin-like GPCR gene expression in Bupivacaine HCl-injected, DMSO-injected and no-injected mosquitoes. As expected, cAMP production was indeed inhibited with Bupivacaine HCl injection, leading to significantly reduced expression of all four P450s (*P* values ranged from 0.045to <0.001) and both PKA genes (*P* values ranged from 0.015 to 0.008), as well as increased mortality of mosquitos to permethrin (P < 0.001) compared with DMSO- and no-injected mosquitoes (Fig. 6). No significant reduction in the levels of GPCR expression was observed in the Bupivacaine HCl-injected mosquitoes compared with the DMSO- and no-injected mosquitoes (Fig. 6). These results strongly suggest that the GPCR regulated cAMP pathway is involved in both P450 gene expression and P450-mediated resistance.

## Discussion

This study explored the regulatory mechanisms of a rhodopsin-like GPCR and its signaling pathway underlying the up-regulation of cytochrome P450 genes associated with insecticide resistance in the mosquito *Cx. quinquefasciatus*. P450s are critical for the detoxification and/or activation of xenobiotics such as insecticides. Up-regulation of P450 genes can significantly affect the disposition of xenobiotics in organisms, thus altering their pharmacological/toxicological effects^17^, and insect P450s are known to be important for insecticidedetoxification. Increasing the metabolism of insecticides into less harmful substances and facilitating insecticide excretion by cytochrome P450s are known to play critical roles in allowing insects to defend themselves against insecticides. Indeed, the latest research has revealed *trans* and/or *cis* regulation to be key factors in resistance P450 gene up-regulation. Nevertheless, the regulatory genes/factors in insecticide resistance have yet to be determined and to date none of the regulatory pathways have been identified. It has been proposed that co-up-regulation could provide fresh insights into altered categories/pathways, thereby aiding functional interpretation^18^. These co-expressed genes are frequently hypothesized to be co-regulated genes^19^. Therefore, characterizing the co-regulated genes in P450s and GPCRs represents a good starting point for efforts to map the entire transcriptional regulatory network. Given a knowledge of the multiple P450 genes and GPCR genes involved in co-up-regulation in the mosquito *Cx. quinquefasciatus*^10^,^11^,^14^,^20^,^21^, we initially extended our research to characterize the function of four up-regulated P450 genes *CYP9M10, CYP9J34, CYP9J40* and *CYP6AA7* and the rhodopsin-like GPCR gene in insecticide resistance, enabling us to examine the relationship between the rhodopsin-like GPCR and the overexpression of resistance P450 genes^6^,^11^,^22^ in insecticide-resistant mosquitoes. Employing a combination of RNAi and/or the *Drosophila* transgenic techniques, functions of the P450 and the rhodopsin-like GPCR genes in mosquito resistance were characterized. Knocking down/reducing the expression of each of the P450 genes and the rhodopsin-like GPCR gene in the resistant mosquitoes was directly linked to decreased levels of resistance to permethrin in resistant strains. The rhodopsin-like GPCR function was further confirmed using transgenic lines of *Drosophila melanogaster*, in which expression of the rhodopsin-like GPCR resulted in an increased level of resistance to permethrin. These results provide strong evidence demonstrating the importance of all four P450 genes and the rhodopsin-like GPCR gene in mosquito resistance.

Given the regulatory function of GPCRs in signal transduction and hence in many of the essential physiological functions of organisms, we hypothesize that insect GPCRs are also involved in the development of insecticide resistance through their regulation of P450 gene expression. To test our hypothesis, we examined expression of four of the up-regulated P450 genes in rhodopsin-like GPCR knockdown mosquitoes and discovered that reducing levels of this GPCR led directly to reduced expression of all four P450 genes, strongly suggesting that the rhodopsin-like GPCR is indeed a regulatory factor for P450 gene expression. Furthermore, *Drosophila* transgenic lines containing the mosquito rhodopsin-like GPCR were used to investigate the possibility that GPCR-mediated signaling may represent a general regulatory mechanism of P450 expression across insects. In these lines, characterization of the expression of *Drosophila* P450 genes (*CYP12d1* and *CYP6a8*) that have been implicated in insecticide resistance^15^ revealed that signaling by the mosquito GPCR significantly increased their expression levels, while corresponding to increased levels of resistance. Together, these findings not only demonstrate the importance of the rhodopsin-like GPCR in permethrin resistance, but also suggest, for the first time, that rhodopsin-like GPCR is a general regulator of resistance P450 genes in different insect species.

These novel discoveries will bring renewed attention to efforts to explore the effects of cyclic adenosine 3′,5′-monophosphate (cAMP) and protein kinases (PKAs), the downstream effectors in the GPCR regulatory pathways^23^, and their impact on the regulation of P450 gene expression in mosquitoes and in insecticide resistance in insects generally. Indeed, the AMP-active protein kinase (PKA) regulated-signal transduction pathway has been found to be involved in the regulation of P450 gene expression in many other organisms, ranging from human beings^24^ to chick cells^25^. Bhaskara *et al.*^26^ reported that the caffeine-inducible promotion of *Cyp6a* genes is probably regulated through the cAMP pathway in *D. melanogaster*. Activation or inhibition of an AMP-activated protein kinase could increase or decrease P450 4F2 expression, respectively, in the human hepatocyte cell line^24^. The protein kinase A (PKA) regulated-signaling pathway also plays a crucial role in P450 expression in mouse and rat tumor cells^27^. The RNAi functional tests performed for the current study revealed that knockdown of the rhodopsin-like GPCR gene in resistant mosquitoes resulted in a simultaneous reduction in the expression of protein kinase A genes (PKA000798 in HAmCq^G8^ and MAmCq^G6^), accompanied by a simultaneous reduction in mosquitoes’ resistance to permethrin. Knockdown of the PKA genes in resistant mosquito strains, on the other hand, decreased the expression levels of P450 genes, but not the rhodopsin-like GPCR gene. Inhibition of the cAMP-dependent protein kinase PKA pathway with the cAMP product-specific inhibitor Bupivacaine hydrochloride suppressed the expression of both PKA and the P450 genes, while decreasing the level of resistance in mosquitoes.

Taken together, this study, for the first time, provides strong evidence that the GPCR intercellular pathway is involved in the development of resistance to permethrin in *Cx. quinquefasciatus* and acts by regulating the expression of resistance-related cytochrome P450 genes through the cAMP-dependent protein kinase A (PKA) pathway. This discovery provides a framework and new avenue to characterize further the GPCRs and their regulatory pathways in mosquitoes and other insect species and to elucidate their functions in the regulation of resistance P450 genes in general.

## Methods

### Culex quinquefasciatus Mosquito strains

Five mosquito strains of *Culex quinquefasciatus* were used in this study. S-Lab is an universal insecticide susceptible strain obtained from Dr. Laura Harrington (Cornell University, Ithaca, NY); HAmCq^G0^ and MAmCq^G0^ are field parental resistant strains collected in 2002 from Huntsville and Mobil, respectively, both in Alabama, USA^28^. HAmCq^G0^ and MAmCq^G0^ were established in the laboratory without further exposure to insecticides. The resistance level to permethrin in HAmCq^G0^ and MAmCq^G0^ is 10-fold that in with S-Lab^20^. The HAmCq^G8^ and MAmCq^G6^ strains are the 8^th^ and 6^th^ generations of permethrin-selected HAmCq^G0^ and MAmCq^G0^ offspring, respectively, and have 2,700- and 590-fold higher levels of resistance than S-Lab, respectively^20^. These two strains have been maintained in the laboratory with biannual selection with permethrin and their resistance levels re-measured every three months. All mosquitoes were reared at 25 ± 2 °C under a photoperiod of 12:12 (L:D) h. All the mosquito strains were reared strictly under identical rearing conditions to ensure all five entered into the fourth instar stage at the same time, which was achieved through controlling the egg raft collection, egg hatching, and subsequent larval development and sample collection.

### RNA extraction, cDNA preparation, and the 3′ and 5′ race

A total of 40 4^th^ instar larvae and 2- to 3-day-old adults from each of the 5 mosquito strains (HAmCq^G0^, HAmCq^G8^, MAmCq^G0^, MAmCq^G6^ and S-Lab) had their RNA extracted for each experiment using the acidic guanidine thiocyanate-phenol-chloroform method^9^. The RNA extraction from each strain was performed three times with different mosquito samples on different days to provide biological replications for the northern blot analysis and, later, as the replications of qRT-PCR experiments for the validation of gene expression, when once again three replications were performed, each on a different day. Ten micrograms of RNAs was treated with TURBO DNA-*free*™ DNase (Ambion). Rapid amplification of 3′ and 5′cDNA ends (3′and 5′-RACE) was carried out using the Marathon^TM^ cDNA Amplification Kit (Clontech)^29^. The first strand cDNAs were synthesized with AMV reverse transcriptase using mosquito mRNAs as templates. The double strand cDNA was synthesized following the protocol described by the manufacturer (Clontech). Adaptors were ligated to both ends of the double strand cDNA as described by the manufacturer. The double strand cDNAs were amplified by PCR with the primers (RhoS1 and RhoAS1, Table S1) designed according to our previous rhodopsin-like GPCR EST sequence^11^ (EC093819). The resulting full length PCR product was purified using QIAquick Gel Extraction Kit (Qiagen). The purified PCR products were ligated into the pCR 2.1 vector using the Original TA Cloning kit (Invitrogen) as described by the manufacturer. The full length of the rhodopsin-like gene was cloned in a One Shot TOPO 10F’ cell using the One Shot TOP10F’ Chemically Competent *E. coli* kit (Invitrogen) and grown in LB plate (Kan.^+^). The plasmid was extracted using the EndoFree plasmid Maxi Kit (Qiagen) following the manufacturer’s instructions and sequenced. The full length of the rhodopsin-like GPCR gene was amplified from the cDNA of *Cx. quinquefasciatus* using platinum Taq DNA polymerase High Fidelity (Invitrogen) with a specific primer (RhoS2 Table S1) according to the 5’end sequence of the GPCR 5’Race and oligo(dT) primer^30^ (Table S1). The full length PCR product was then purified, cloned and sequenced. Cloning and sequence analyses of the rhodopsin-like GPCR full length cDNAs were repeated at least three times with different preparations of RNAs, and three TA clones from each replication were verified by sequencing.

### Quantitative Real-time PCR (qRT-PCR)

Total RNA samples (0.5 μg/sample) from larval and adult mosquitoes were reverse-transcribed using SuperScript II reverse transcriptase (Stratagene) in a total volume of 20 μl. The quantity of cDNAs was measured using a spectrophotometer prior to qRT-PCR, which was then performed with the SYBR Green master mix Kit and ABI 7500 Real Time PCR system (Applied Biosystems). Each qRT-PCR reaction (25 μl final volume) contained 1× SYBR Green master mix, 1 μl of cDNA, and a GPCR gene specific primer pair (designed according to each of the gene sequences; Table S1) at a final concentration of 3–5 μM. All samples, including a ‘no-template’ negative control, were performed in triplicate. The reaction cycle consisted of a melting step of 50 °C for 2 min then 95 °C for 10 min, followed by 40 cycles of 95 °C for 15 sec and 60 °C for 1 min. Specificity of the PCR reactions was assessed by a melting curve analysis for each PCR reaction using Dissociation Curves software^31^. Relative expression levels for P450 genes were calculated by the 2^−ΔΔCT^ method using SDS RQ software^32^. The 18S ribosome RNA gene, an endogenous control, was used to normalize the expression of target genes^33^,^34^. Preliminary qRT-PCR experiments with the primer pair (Table S1) of 18S ribosome RNA gene designed according to the sequences of the 18S ribosome RNA gene had revealed that the 18S ribosome RNA gene expression remained constant among all 5 mosquito strains, so the 18S ribosome RNA gene was used for internal normalization in the qRT-PCR assays. Each experiment was repeated three times with different preparations of RNA samples.

The statistical significance of the gene expression was calculated using a Student’s t-test for all 2-sample comparisons and a one-way analysis of variance (ANOVA) for multiple sample comparisons using Statistical Package for the Social Sciences (SPSS) software with both Least Significant Difference (LSD) and Tukey tests to analyze the significance of means. A value of P ≤ 0.05 was considered statistically significant. Significant up-regulation or down-regulation was determined using a cut-off value of a “2-fold change in expression”^35^.

### Northern Blot Analysis

Northern blot analyses were performed according to Sambrook *et al.*^36^. Five micrograms of mRNA from 4^th^ instar larvae and 2-3-day old adults of 5 mosquito strains were fractionated on 1% formaldehyde denaturing agarose gel containing ethidium bromide and transferred to Nytran membranes. The rhodopsin-like GPCR gene (~500bp) was amplified using a Northern RhoF and Northern RhoR primer pair (Table S1). The PCR product of the rhodopsin-like GPCR gene was labeled with [α-^32^P]dCTP using a Prime-It II Random Primer Labeling kit (Agilent Technologies, Stratagene) following the manufacturer’s instructions, and hybridized with RNA blots using QuickHyb solution (Agilent Technologies, Stratagene). The quantity of RNA loaded in each lane was standardized by comparing the density of the 18S ribosomal RNA (rRNA) band on the agarose gel under UV light before transfer^37^. All Northern blot analyses were repeated three times with different preparations of RNA samples. The statistical significance of the gene expression was calculated as described in section Quantitative Real-time PCR (qRT-PCR).

### Construction of Double-stranded RNA (dsRNA) *in vitro*

For the initial investigation of the function of the rhodopsin-like GPCR, PKA, and P450 genes in *Culex* mosquitoes, we used the double-stranded RNA interference (RNAi) technique, a powerful tool to silence the gene expression post-transcriptionally^38^. Briefly, an approximate ~250–650 bp fragment from each of the GPCR, PKA, and P450 genes and a green fluorescent protein (GFP gene – pMW1650, generously provided by Dr. Z. Xi, Michigan State U.) were generated that were complementary to the cDNA sequences of the genes and pMW1650 plasmid, respectively, with PCR, using each gene-specific primer pair with T7 promoter sequences (5′-TAATACGACTCACTATAGGG-3′) appended at the 5′ end of each PCR primer (Table S1). dsRNA was synthesized from the PCR template with the opposing T7 promoter sequences by *in vitro* transcription using the MEGAscrip T7 High Yield Transcription kit (Life Technologies), following the manufacturers’ instructions. The dsRNAs were then purified by phenol/chloroform extraction followed by ethanol precipitation.

### Embryonic Injection

The function of the rhodopsin-like GPCR, PKA and P450 genes in larvae of the two highly resistant mosquito strains, HAmCq^G8^ and MAmCq^G6^, was tested independently by injection of the rhodopsin-like GPCR, PKA, or P450 dsRNA into mosquito embryos^39^,^40^,^41^. Briefly, ~1000 fresh grey embryos were collected from each of HAmCq^G8^ and MAmCq^G6^ for each dsRNA injection. Batches of around 120 embryos at a time of each strain were arranged on a piece of paper filter and allowed to dry for 2-3 min and then transferred to a microscope cover slide (VWR Scientific Products) using double sided tape and covered with Halocarbon 700 oil (Halocarbon Products Corp.) against dehydration. A glass capillary (Borosil 1.0 mm ODx0.5 mm ID/Fiber, 100 mm length, FHC, Inc) was pulled using a Model P-97 Flaming/Brown micropipette puller (Sutter Instrument. Co.), following the program: Heat 525, Vel 50, Time 250, and Pull 50. The glass needle tip was opened using a Model BV-10, K. T. Brown Type micro-pipette beveller (Sutter Instrument, Co.). After injecting 0.2–0.5 nl of dsRNA at a concentration of 3.5 ug/ul into each embryo posterior horizontally using a Picospritzer III injector system (Parker Instrumentation) under the Nikon Eclipse TS100 microscope (Nikon Instruments), the injected embryos were transferred into clean water and kept at 25 ± 2 °C under a photoperiod of 12:12 (L:D) h for 3 days while they developed into the second instar larvae. The hatched 2^nd^ instar larvae of the dsRNA injected mosquitoes were separated into two groups of ~360 each, one of which was tested for permethrin insecticide bioassay and the other prepared for extracting the RNA to test the gene expression using qRT-PCR. Mosquitoes that were injected with GFP dsRNA served as the controls and those receiving no injection were the calibrators. Mosquitoes that were injected with non-differential expressed gene(s) served as a negative control to guard against false positive results caused by the indirect effect of silencing GPCR, which could affect the ability of the insects to increase their response to an insecticide in general. Each experiment was repeated more than 3 times with independently isolated RNA samples.

### Permethrin insecticide bioassay on mosquitoes

Stock and serial dilutions of permethrin (94.34%, supplied by FMC Corp., Princeton, NJ) for the insecticide bioassays were prepared in acetone. The bioassay method used for the larvae was as described in previous studies^20^. Each bioassay consisted of 4^th^ instar mosquito larvae of rhodopsin-like GPCR, P450, PKA-dsRNA, GFP-injected or non-dsRNA-injected mosquitoes in regular tap water and 1% insecticide solution in acetone at the required concentration, using four to eight concentrations that resulted in >0 and <100% mortality. Larva bioassay was conducted at 3-days after dsRNA of different P450 gene injection (2^nd^ instar larvae). LC_50_ was analyzed using standard probit analysis. Control groups received only 1% acetone. Mortality was assessed after 24 h. All tests were run at 25 °C and each assay was replicated at least 3 times. Bioassay data were pooled and analyzed by standard probit analysis, as described by Liu *et al.*^28^, utilizing a computerized version of Raymond^42^.

The statistical analysis for insecticide bioassay was conducted by examining the LC_10_, LC_50,_ and LC_90_ values based on non-overlapping 95% confidence intervals (CI). Resistance ratios (RRs) were calculated by dividing the LC_50_ of the specific gene injected or GFP-injected mosquitoes by the LC_50_ of the no-injected mosquitoes.

### Construction of transgenic *Drosophila* flies

The rhodopsin-like GPCR full length cDNA from the TA clone was amplified using primer pair of Rho F and Rho R designed based on the 5′ and 3′ end sequences of the rhodopsin-like GPCR gene, respectively, with protecting bases CCG and restriction enzyme cutting site EcoRI sequence (GAATTC) in RhoF and CTA and Xbal sequences (TCTAGA) in Rho R (Table S1). The rhodopsin-like GPCR gene was then sub-cloned into a pUASTattB vector (a gift from Dr. Johannes Bischof, University of Zurich). The plasmids of the pUASTattB- rhodopsin-like GPCR gene and the empty vector of pUASTattB (used as a control) were transformed into the germ line of the M{vas-int.Dm}ZH-2A, M{3xP3-RFP.attP’}ZH-58A strain of *D. melanogaster* (Bloomington stock #24484), resulting in site specific integration on chromosome 2R (Rainbow Transgenic Flies Inc., Camarillo, CA). Flies were then reciprocally-crossed against a W^1118^ strain to obtain transformants with the orange eye phenotype, then balanced against the *D. melanogaster* balancer strain w[1118]/Dp(1;Y)y[+]; sna[Sco]/CyO, P{ry[+t7.2] = sevRas1.V12}FK1 (Bloomington stock #6312) to generate a homozygous line containing the rhodopsin-like GPCR transgene. The insertion of the rhodopsin-like GPCR transgene into the transgenic fruit fly lines was further confirmed using RT-PCR. The resulting homozygous transgenic virgin female *D. melanogaster* line was then crossed with the GAL4-expressing *D. melanogaster* strain P{Act5C-GAL4}17bFO1 (Bloomington stock #3954), which expresses GAL4 under control of the Act5C promoter, resulting in ubiquitous non-tissue-specific expression. The F1 generation of these crosses expressed GAL4 and contained a single copy of the rhodopsin-like GPCR transgene, which was under the control of the UAS enhancer. All *D. melanogaster* were reared on Jazz-Mix Drosophila food (Fisher Scientific, Kansas City, MO) at 25 ± 2 °C under a photoperiod of 12:12 (L:D) h.

### Toxicity of permethrin on the transgenic lines of *D. melanogaster*

Permethrin toxicity bioassays were then conducted on 2–3 day post eclosion female *Drosophila* of F1 UAS-GAL4 crosses to examine the toxicity of permethrin to transgenic flies. Briefly, serial concentrations of permethrin solution in acetone, ranging from 3 ng/μL to 100 ng/μL, that resulted in >0 and <100% mortality for the tested insects were prepared, after which 200 μL of each permethrin solution was evenly coated on the inside of individual 20 ml glass scintillation vials. Fifteen female flies were transferred to each of the prepared vials, which were plugged with cotton balls soaked with 5% sucrose. The vials for the control groups were coated with acetone alone and plugged with identical 5% sucrose-soaked cotton balls. The mortality was scored after 24 hr exposure to permethrin. Once the concentration range had been determined, a single concentration (50 ng/μL) that showed a significant difference in mortality between the transgenic flies with rhodopsin-like GPCR gene and the vector flies was selected for further permethrin bioassays. For each bioassay, a total of four vials containing fifteen 2-3-day old adult female *D. melanogaster* each were treated with 200 μL of 50 ng/μL permethrin solution for each strain. Each bioassay was independently replicated three times using only flies that exhibited the correct morphological marker (red eyes). The *D. melanogaster* strain M{vas-int.Dm}ZH-2A, M{3xP3-RFP.attP’}ZH-58A containing the empty pUAST vector donated insert but no transgene from *D. melanogaster* was used as the control reference strain. Mortality was assessed after 24 h.

The statistical analysis for average survival values of each bioassay was conducted using a Student’s t-test for all 2-sample comparisons and a one-way analysis of variance (ANOVA) for multiple sample comparisons using SPSS software with both LSD and Tukey tests to analyze the significance of the means; a value of P ≤ 0.05 was considered statistically significant.

### Larva injection with cAMP production inhibitor- Bupivacaine hydrochloride

The larva injection method was also used to inject the cAMP production inhibitor, Bupivacaine HCl (C_18_H_28_N_2_O.HCl), (Selleckchem) into 3^rd^ instar larvae of HAmCq^G8^ and MAmCq^G6^. Briefly, the microinjection glass needle was pulled by the Borosilicate Glass Capillary (1 mm ODx0.58 ID mm, 100 mm length, World Precision Instruments, Inc.) using Model P-97 Flaming/Brown micropipette puller (Sutter Instrument. Co.) following the program: Heat 454, Vel 120, Time 125, and Pull 30. The Nanoinject II injector (Drummond Scientific Company) was used to pull Bupivacaine HCl in dimethyl sulfoxide (DMSO) into the glass needle for injection. The individuals were transferred from clean water to dry filter paper to immobilize the larvae and each was injected with 69 nl of 40 μM inhibitor in Dimethyl sulfoxide (DMSO) vertically into the body axis between the thorax and abdomen twice, for a total of ~138 nl. Injections of DMSO alone served as the control and non-injection served as the calibrator. To make the injection visible, 0.05% phenol red solution (Sigma) was added to both the inhibitor and the DMSO alone. After the injection, the larvae were immediately moved from the filter paper back into distilled water and reared under insectary conditions for 72 hrs. The surviving larvae were collected and separated into two groups, one of which was used for the gene expression test, and the other for the larva bioassays with permethrin dose ranges as in the above description. All the experiments were replicated more than three times with independent injection and sample collection. The statistical analysis for insecticide bioassay was conducted as described in the section of permethrin insecticide bioassay on mosquitoes above.

## Additional Information

**How to cite this article**: Li, T. *et al.* A G-protein-coupled receptor regulation pathway in cytochrome P450-mediated permethrin-resistance in mosquitoes, *Culex quinquefasciatus. Sci. Rep.* **5**, 17772; doi: 10.1038/srep17772 (2015).

## Supplementary Material

Supplementary Information
