# Supplementary material for: A G-protein-coupled receptor regulation pathway in cytochrome P450-mediated permethrin-resistance in mosquitoes, Culex quinquefasciatus
Source: Sci Rep. 2015 Dec 10;5:17772. doi: 10.1038/srep17772 (PMC4674712; doi:10.1038/srep17772)
Supplement: Supplementary Information [file srep17772-s1.pdf]

Scientific Reports

A G-protein-coupled receptor regulation pathway in cytochrome P450-mediated permethrin-resistance in mosquitoes, *Culex quinquefasciatus*

Ting Li[1], Chuanwang Cao[1,4], Ting Yang[1,5], Lee Zhang[2], Lin He[1,6], Zhiyong Xi[3], Guowu Bian[3,7], and Nannan Liu[1*]

[1]Department of Entomology and Plant Pathology, Auburn University, Auburn AL 36849 [2]Genomics Laboratory, Auburn University, Auburn, AL 36849

[3]Department of Microbiology and Molecular Genetics, Michigan State University, East Lansing, MI 48824

[4]Current address: College of Forestry, Northeast Forestry University, Harbin, China

[5]Current address: Institute of Plant Protection, Chinese Academy of Agricultural Sciences, Beijing, China

[6]Current address: College of Plant Protection, Southwest University, Chongqing, China

[7]Current address: Zhongshan School of Medicine, Sun Yat-Sen University, China

[1*]Correspondence should be addressed to: Dr. Nannan Liu, 301 Funchess Hall, Auburn University, Auburn, AL 36849, USA. Phone: (334) 844-2661, E-mail: liunann@auburn.edu

Key words: Mosquito vector; insecticide resistance; G-protein coupled receptor; Protein Kinase A; GPCR/cAMP/PKA signal transduction pathway; gene regulation

Table S1. Oligonucleotide primers used in qRT-PCR and PCR reactions

Primer Description	Primer Name	Primer Sequence
18S Ribosomal RNA	18S rRNA F1	5'CGCGGTAATTCCAGCTCCACTA3'
	18S rRNA R1	5'GCATCAAGCGCCACCATATAGG3'
3' and 5' Race Rhodopsin-like gene amplification	Rho S1	5'CCTTCTCGGACTTCCTGAT3'
	Rho AS1	5'AGCCGAACAGTGATCCACAC3'
	Rho S2	5'GAGCTCTAACAAGTCCATCCAAG3'
	Oligo(dT)	5'TAATACGACTCACTATAGGGAGATTTTTTTTTTTTTTTT3'
Drosophila construction	RhoF	5'CCGGAATTCCAAAATGGCATCTTACGCAGCATGGAC3'
	RhoR	5'CTAGTCTAGAGGCCTTCTCGTCCGAAGCG3'
Rho.-like GPCR Northern blot analysis	Northern RhoF	5'GGGCCATCTTCTTCCTGTGC3'
	Northern RhoR	5'GCGGGGCGAAGTACACGAAC3'
Rho.-like GPCR Real-time PCR	qPCR iRho F	5'ACTACCTCACCGACACCTTCTC3'
	qPCR iRho R	5'GCCTTGATGATGAAGATG3'
Rho.-like GPCR dsRNA synthesis	dsRNA RhoF	5'TAATACGACTCACTATAGGGGCCATCTTCTTCCTGTGC3'
	dsRNA RhoR	5'TAATACGACTCACTATAGGGCGGGGCGAAGTACACGAA3'
GPCR003158 Real-time PCR	qPCR003158F	5'TGCTTCCTGATTTGCTTCATGCCG3'
	qPCR003158R	5'GCTTTGTGTACGCGTGCCTGTAAT3'
GPCR003158 dsRNA synthesis	dsRNAGPCR003158F	5'TAATACGACTCACTATAGGGTTACACACCAATCAGTCTGGCC3'
	dsRNAGPCR003158R	5'TAATACGACTCACTATAGGGATCAGCGGCATGAAGCAAATCA3'
PKA000798 Real-time PCR	qPCRPKA000798F	5'TTGATTGGTGGGCATTAGGCGTTC3'
	qPCRPKA000798R	5'AGCAGCTTCTTGACCAGGTCCTTT3'
PKA000798 dsRNA synthesis	dsRNAPKA000798F	5'TAATACGACTCACTATAGGGTGAAGCAGATCGAGCACGTCAA3'
	dsRNAPKA000798R	5'TAATACGACTCACTATAGGGAGATGCCGAACGGATTATCGTC3'
PKA018257 Real-time PCR	qPCRPKA018257F	5'ATACCGTGACTTGAAGCCGGAGAA3'
	qPCRPKA018257R	5'AATTTGTATTGGCTGATCAGC3'
PKA018257 dsRNA synthesis	dsRNAPKA18257F	5'TAATACGACTCACTATAGGGCAAGTTGAGCACACCCTAAA3'
	dsRNAPKA18257R	5'TAATACGACTCACTATAGGGCTTCAAGTCACGGTATATCA3'
PKA004656 Real-time PCR	qPCRPKA004656F	5'CATGCACAAGCGCTTCATCGTGTA3'
	qPCRPKA004656R	5'CGTGCGGCTTCTTCTTGCTGAAAT3'
PKA015942 Real-time PCR	qPCRPKA015942F	5'GCGATCGCCAAGAACGTGCTATTT3'
	qPCRPKA015942R	5'GGTCACCTGTTCGCTGTTCACAAA3'
GFP gene dsRNA synthesis	dsRNAGFPF	5'TAATACGACTCACTATAGGGAGAAGAACTTTTCACTGG3'
	dsRNAGFPR	5'TAATACGACTCACTATAGGGCTTCTACCTAGGCAAGTT3'
P450CYP9M10 Real-time PCR (mosquito)	qRTP450-1CxF	5'ATGCAGACCAAGTGCTTCCTGTAC3'
	qRTP450-1CxR	5'AACCCACTCAACGTATCCAGCGAA3'

P450CYP9M10 dsRNA synthesis	dsRNACYP9M10F	5’TAATACGACTCACTATAGGGAACAACGACCGTCATCTGC3’
	dsRNACYP9M10R	5’TAATACGACTCACTATAGGGGCTTGTTCAGCTTGATCGGC3’
P450CYP9J40 Real-time PCR (mosquito)	qRTP450-23CxF	5’ACCCGAATCCGGGCAAGTTTGAT3’
	qRTP450-23CxR	5’AACTCCAAACGGTAAATACGCCGC3’
P450CYP9J40 dsRNA synthesis	dsRNACYP9J40F	5’TAATACGACTCACTATAGGGAGCTAATCAAGAAGGTC3’
	dsRNACYP9J40R	5’TAATACGACTCACTATAGGGATGTCCGGTCGAATGAT3’
P450CYP6AA7 (mosquito)	P4505959F	5’ATGACGCTGATTCCCGAGACTGTT3’
	P4505959R	5’TTCATGGTCAAGGTCTCACCCGAA3’
P450CYP6AA7 dsRNA synthesis	dsRNACYP6AA7F	5’TAATACGACTCACTATAGGGATCGTACTGGGCGGAT3’
	dsRNACYP6AA7R	5’TAATACGACTCACTATAGGGAACAGTCTCGGGAATCA3’
P450CYP9J34 Real-time PCR (mosquito)	P45010546F	5’ATCCGATGTCGGTAAAGTGCAGGT3’
	P45010546R	5’TGTACCTCTGGGTTGATGGCAAGT3’
P450CYP9J34 dsRNA synthesis	dsRNACYP9J34F	5’TAATACGACTCACTATAGGGGGATCGCAACGAAAAG3’
	dsRNACYP9J34R	5’TAATACGACTCACTATAGGGGACACGGTATCAAAACC3’
P450CYP6BY3 Real-time PCR (mosquito)	P4503375F	5’TCCACAAACTTGTAGCCGACACGA3’
	P4503375R	5’GCAGCAGTCTCATCCAGCGTAAGA3’
P450CYP6BY3 dsRNA synthesis	dsRNACYP6BY3F	5’TAATACGACTCACTATAGGGCCGACACGATCGCGTACAG3’
	dsRNACYP6BY3R	5’TAATACGACTCACTATAGGGTCGCAAGTCCGACTCTGGA3’
P450CYP6a2 Real-time PCR (drosophila)	CYP6a2(D)F	5’TGGACGGAAAGAAGTGGAAGGAC3’
	CYP6a2(D)R	5’AGTTCATGTTCCCGACGGTGATCA3’
P450CYP12d1 Real-time PCR (drosophila)	Cyp12d1(D)F	5’GCTCGGCTCAAATGTGCTGATGAA3’
	Cyp12d1(D)R	5’TGACCTGCATCTTCTTTCCGGTCT3’
P450CYP6a8 Real-time PCR (drosophila)	Cyp6a8(D)F	5’ACGAGTGCACCAAGGATCTGAAG3’
	Cyp6a8(D)R	5’ATTGACCAGCCTCGATGACGAAGT3’
P450CYP6g1 Real-time PCR (drosophila)	Cyp6g1(D)F	5’CGGCTGAAGGACGAGGCTGT3’
	Cyp6g1(D)R	5’GCTATGCTGTCCGTGGAGAACTGA3’
